# Supplementary figures and images for: Vacuolar sterol β-glucosidase EGCrP2/Sgl1 deficiency in Cryptococcus neoformans: Dysfunctional autophagy and Mincle-dependent immune activation as targets of novel antifungal strategies
Source: PLoS Pathog. 2025 Apr 24;21(4):e1013089. doi: 10.1371/journal.ppat.1013089 (PMC12061408; doi:10.1371/journal.ppat.1013089)

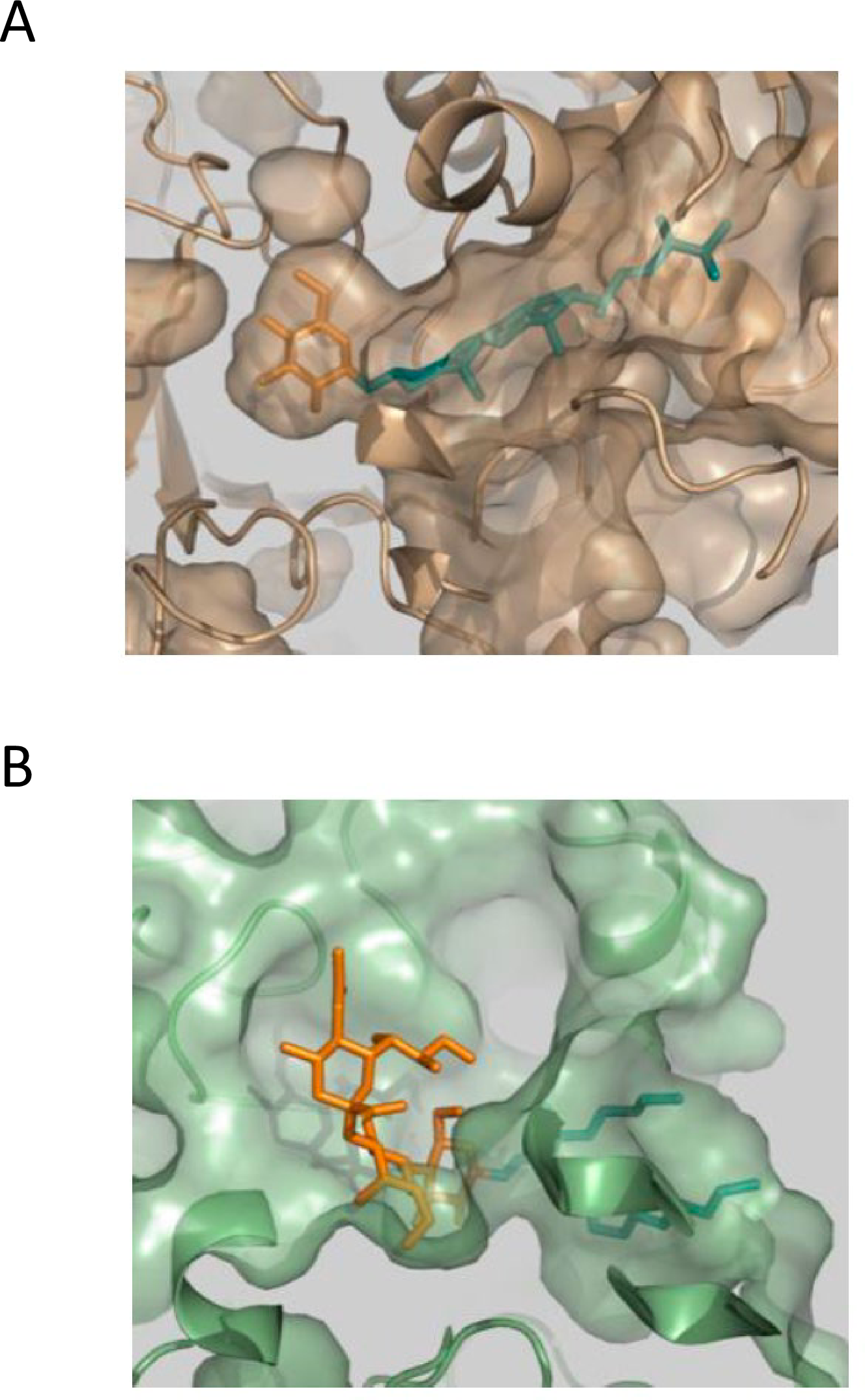

Supplement: S1 Fig — A, Magnified view of the EG binding cleft in the EGCrP2-EG docking model. PDB code: 7LPO. EG can enter the substrate-binding cleft of EGCrP2/Sgl1; however, even lactosylceramide (LacCer), the minimum-sized substrate of EGCase, cannot enter the cleft of EGCrP2/Sgl1. B, Magnified view of the binding cleft of EGCase with GM3 ganglioside (N-acetylneuraminic acid-LacCer). PDB code: 5J7Z. LacCer and glycolipids with longer sugar chains than LacCer, such as GM3, can enter the cleft of EGCase by extending the sugar chain outward from the enzyme. (TIF) [file ppat.1013089.s001.tif]

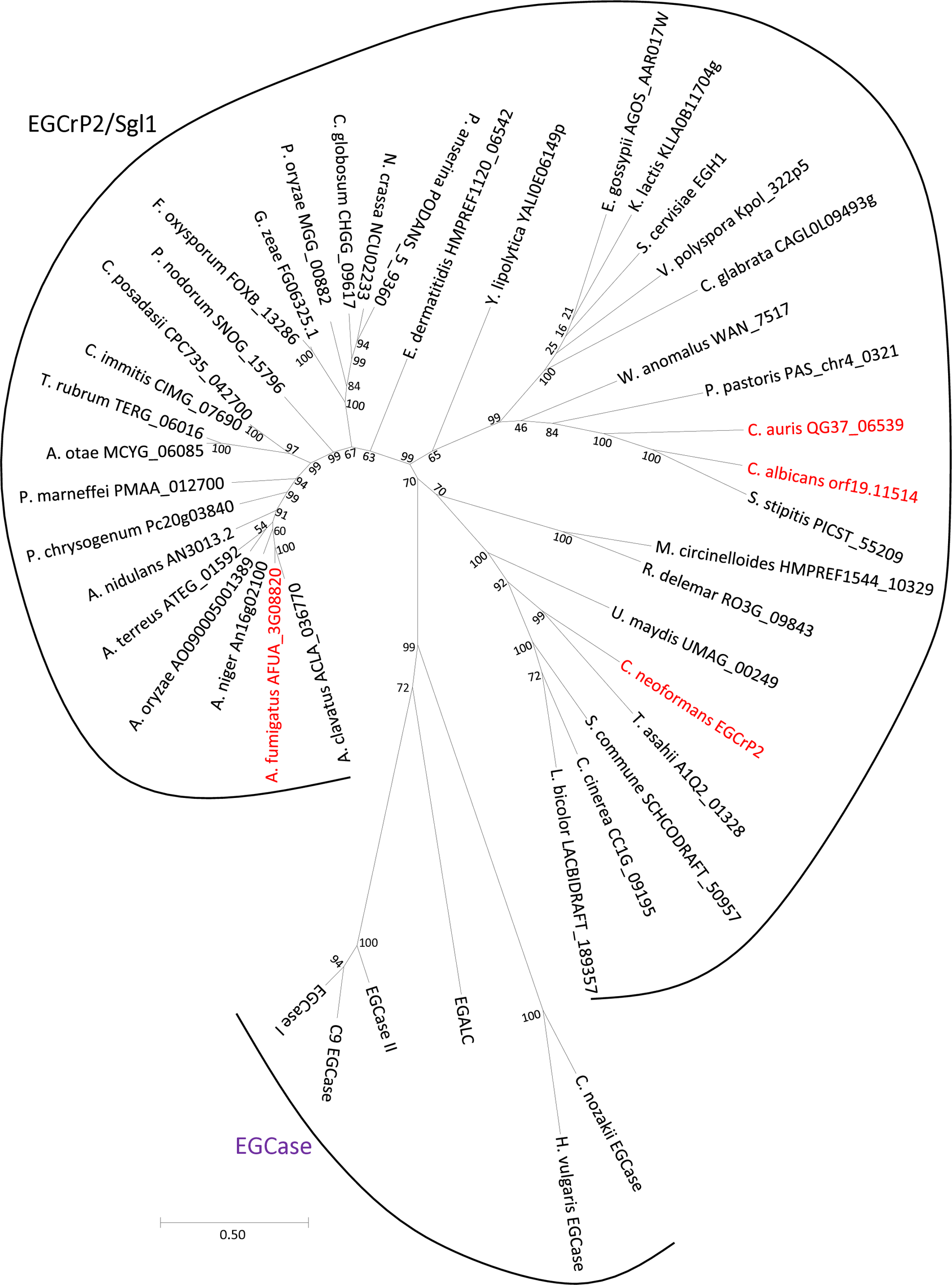

Supplement: S2 Fig — The amino acid sequences of EGCrP2/Sgl1 and related proteins were reconstructed using the neighbor-joining method. The scale bar indicates 0.5 amino acid substitutions per site. The accession number of each protein is listed in S1 Table. Strains in the WHO FPPL critical group are shown in red. (TIF) [file ppat.1013089.s002.tif]

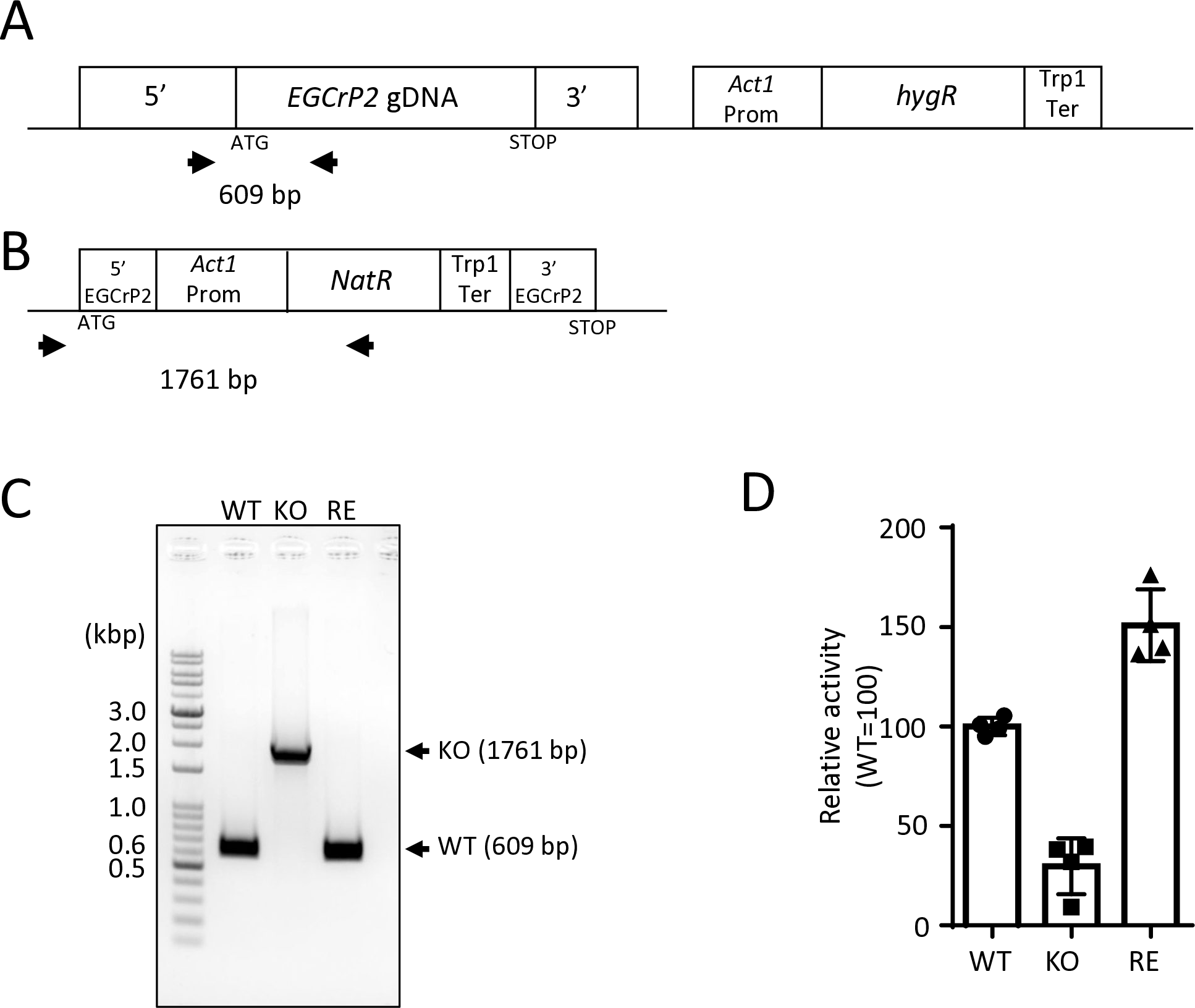

Supplement: S3 Fig — A, SGL1-expressing construct was introduced into the sgl1Δ (KO) strain by targeted integration to be expressed under the control of its original promoter. B, SGL1 KO construct was generated by the method described in [7]. C, PCR analysis of WT, KO, and RE strains. D, EGCrP2/Sgl1 activity of WT, KO, and RE strains. The activity was measured using C12-NBD-GlcCer as a substrate, as described previously [7]. Data are presented as Mean ± SD (n = 4). (TIF) [file ppat.1013089.s003.tif]

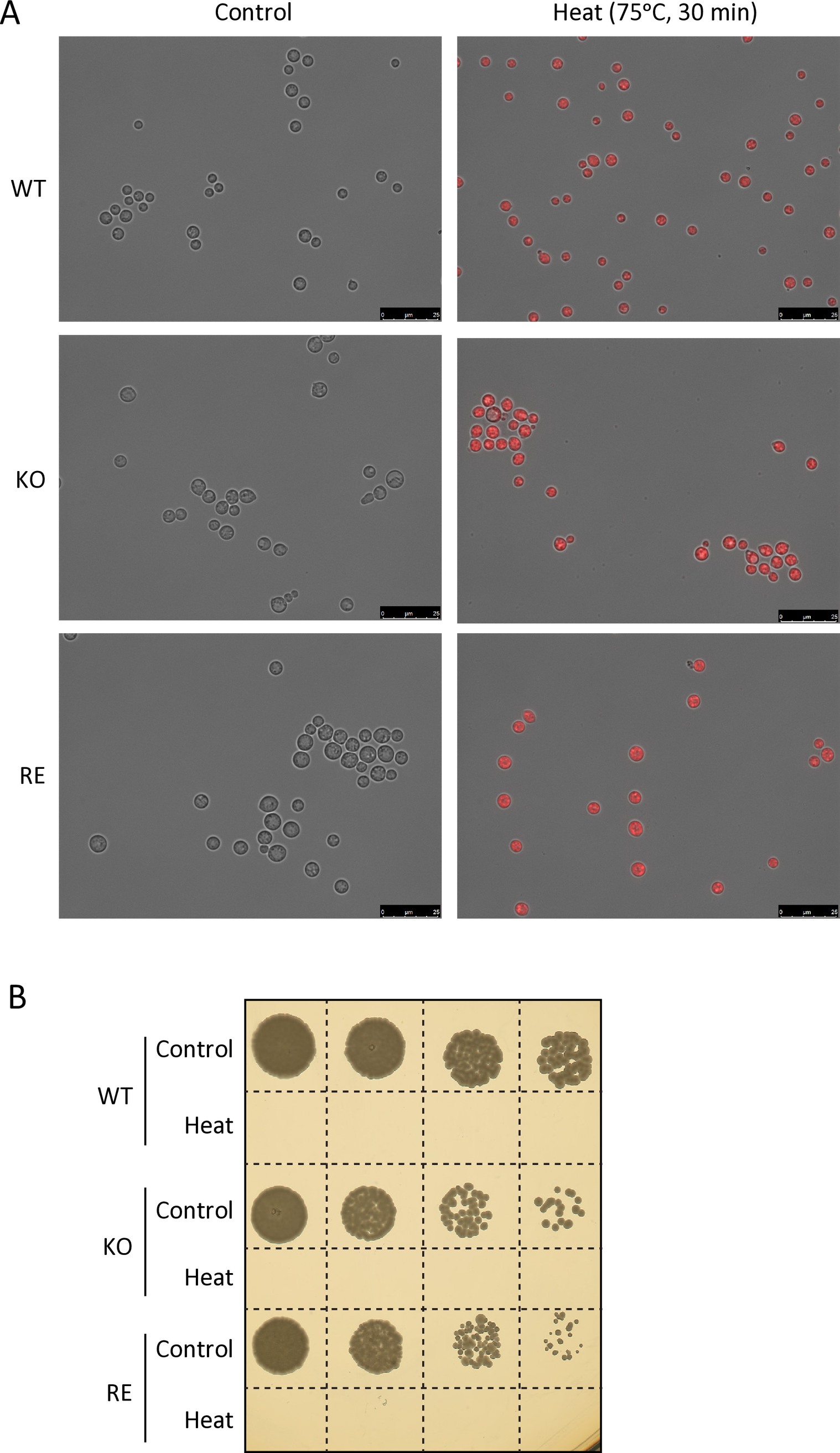

Supplement: S4 Fig — A, Phloxine B staining of control and heat-killed Cn cells. WT (top), KO (middle), and RE (bottom) Cn were incubated at 75°C (heat-killed) or 30°C (control) for 30 minutes before staining with phloxine B. Fluorescence image was captured using a fluorescence microscope with differential interference contrast (DIC) imaging. Scale bar, 25 μm. B, Spot assay of control and heat-killed Cn cells. Tenfold serial dilutions of each strain were spotted onto YPD agar plates and incubated at 30°C for 3 days. (TIF) [file ppat.1013089.s004.tif]

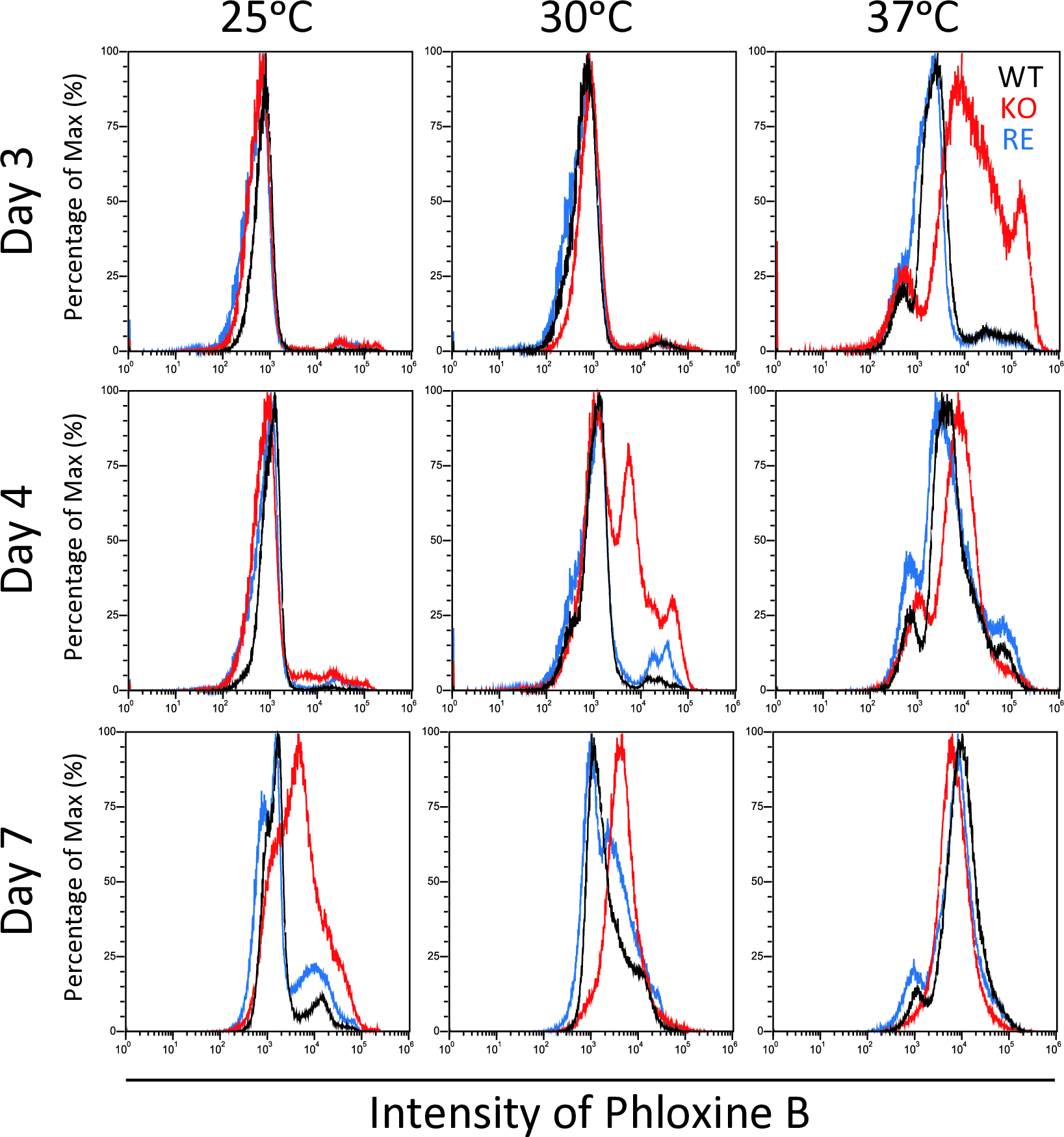

Supplement: S5 Fig — WT, KO, and RE strains were cultured in YPD medium for 3, 4, and 7 days at 25, 30, and 37°C with shaking at 150 rpm. Cells were stained with phloxine B and analyzed by a flow cytometer using a BL2-H channel (574/26 nm BP filter). The data on day 3 was used as Fig 2D. (TIF) [file ppat.1013089.s005.tif]

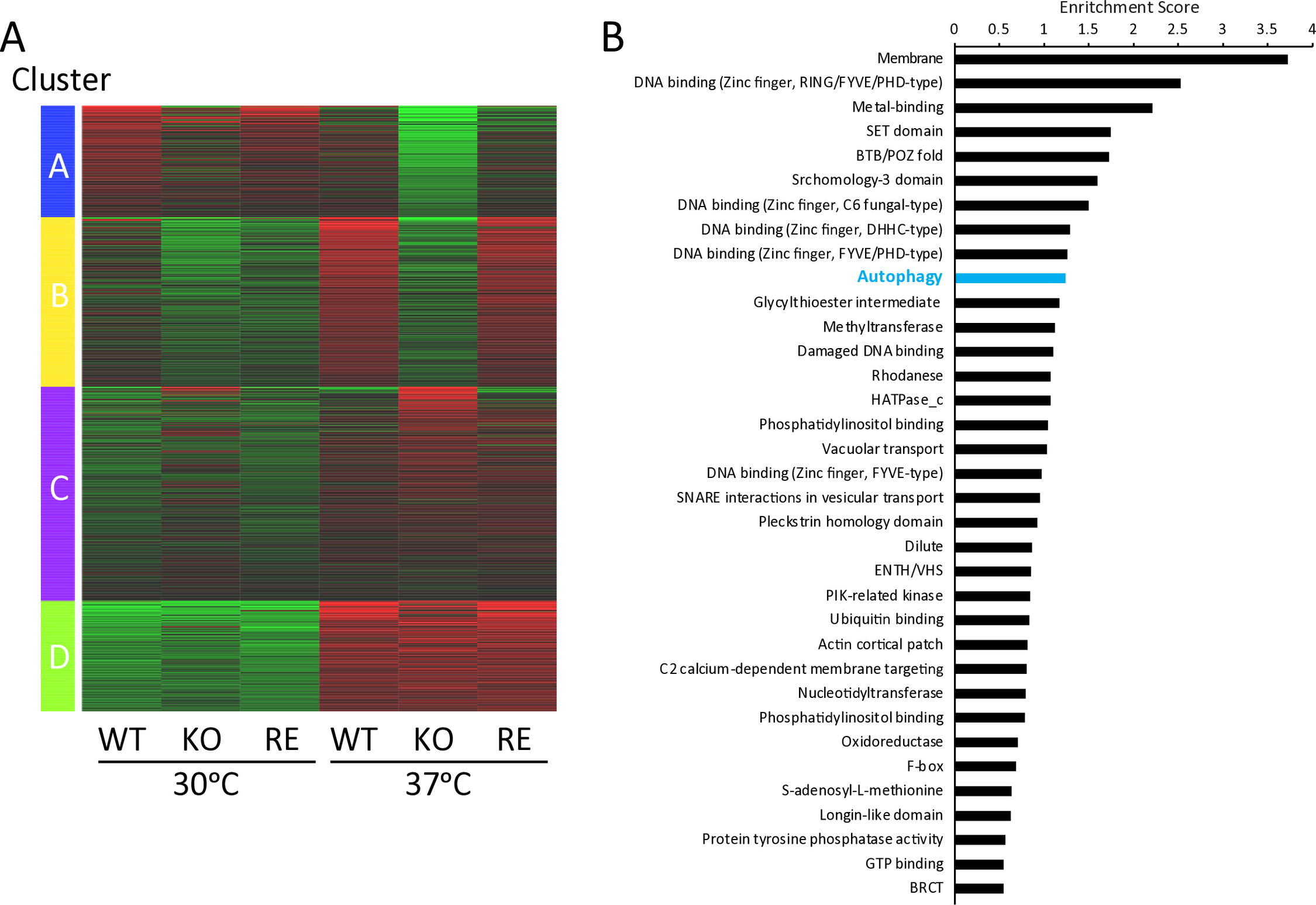

Supplement: S6 Fig — A, K-means clustering of total genes and their expression patterns. RNAseq was performed using total RNA isolated from Cn after 72 h (day 3) of incubation at 30 and 37°C (n = 1 for each WT, KO, RE strains at both temperatures). The heat map shows the relative expression levels of each transcript (rows) in each sample (column). Normalized transcripts per million (TPM) were log 2-transformed and median-centered by transcript. The heatmap was drawn based on clustering results. Red and green colors represent higher and lower expressions, respectively. B, Enrichment scores of gene ontology (GO) terms for genes upregulated in KO strain in Cluster B on DAVID analysis [53]. The enrichment scores for each GO term are displayed in the graph. (TIF) [file ppat.1013089.s006.tif]

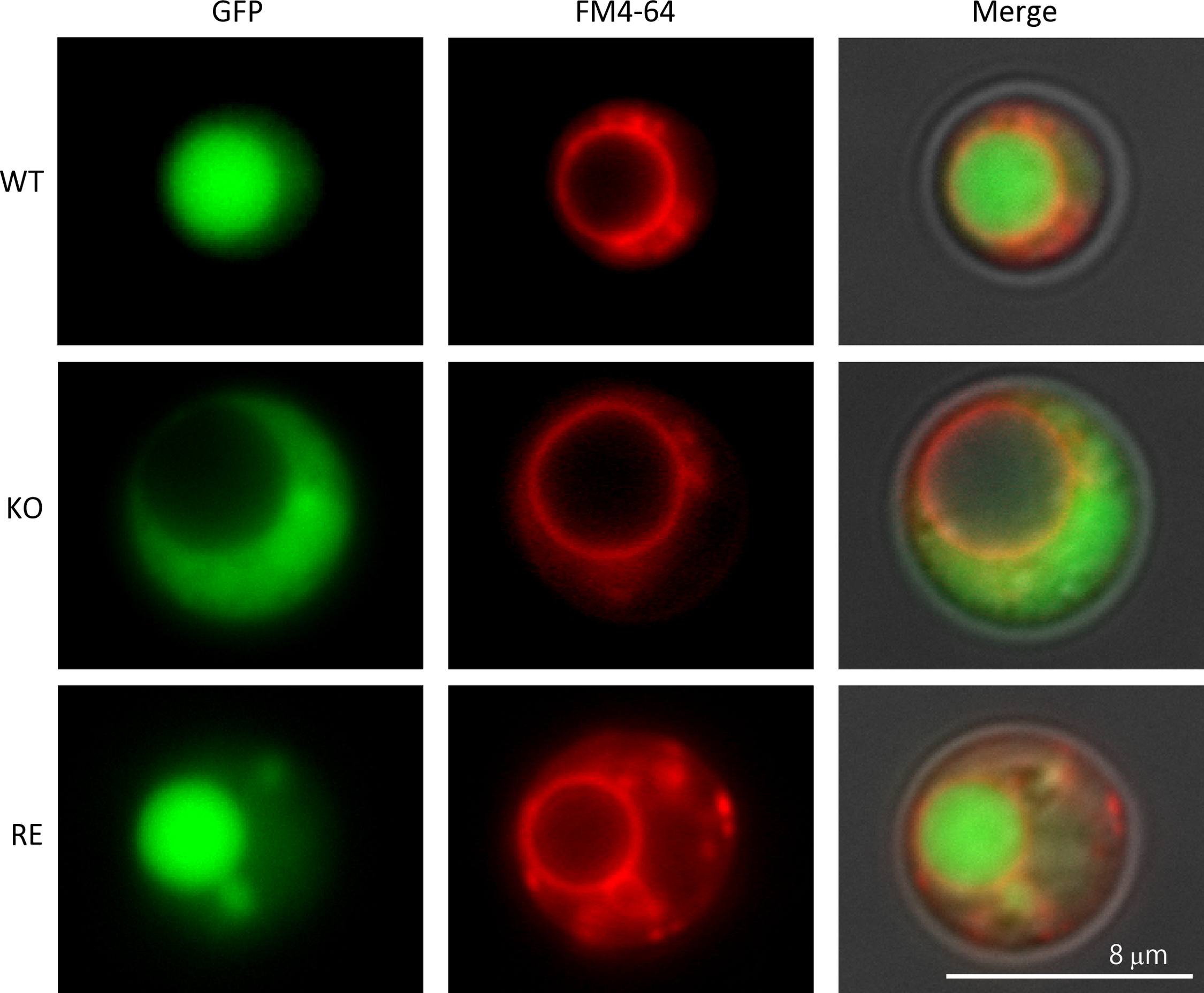

Supplement: S7 Fig — Vacuoles of GFP-Atg8 expressing WT, KO, and RE strains were stained with 16 µ M of FM4–64 for 1 hour. A fluorescence microscope captured GFP (green) and F4-64 (red) images. Scale bar, 8 μm. (TIF) [file ppat.1013089.s007.tif]

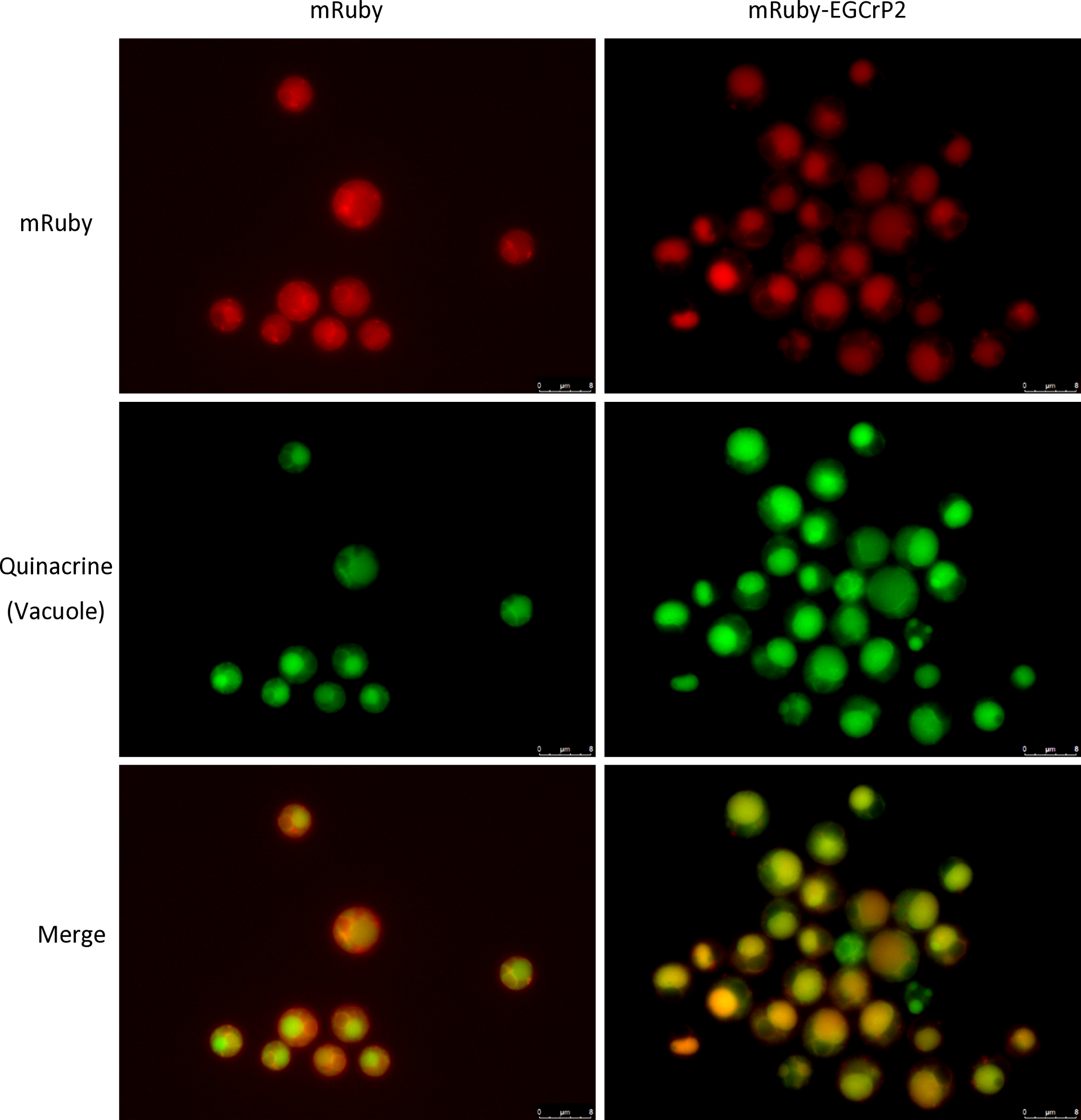

Supplement: S8 Fig — Fluorescence microscopy images show mRuby-derived fluorescence (red), indicating the distribution of mRuby-tagged EGCrP2/Sgl1 (mRuby-EGCrP2/Sgl1) and free mRuby (mRuby). Vacuoles were stained with quinacrine (green), which specifically accumulates in fungal vacuoles [56]. Scale bar, 8 μm. (TIF) [file ppat.1013089.s008.tif]

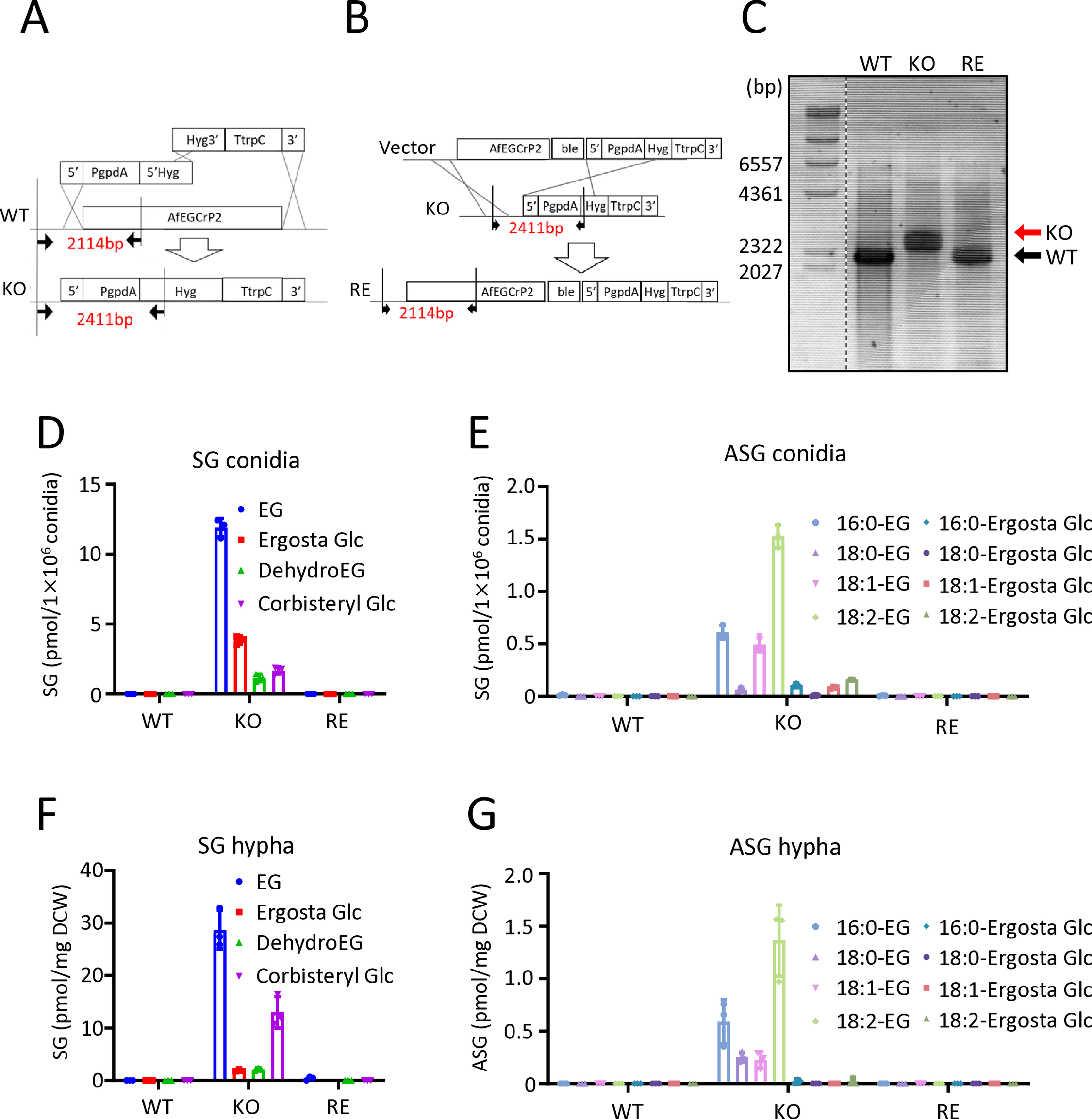

Supplement: S9 Fig — A, Generation of SGLA KO A. fumigatus (sglaΔ, KO) by split-marker method as described in [60]. B, Generation of SGLA revertant A. fumigatus (sglaΔ::SGLA, RE) by targeted integration. C, PCR analysis of WT, KO, and RE strains. D and E show the contents of SGs and ASGs, respectively, in conidia of WT, KO, and RE strains. A. fumigatus was cultured on YPD agar plates at 37°C, harvested, and disrupted using a bead crusher to obtain conidia. F and G show the contents of SGs and ASGs, respectively, in the hyphae of WT, KO, and RE strains. Hyphae of WT, KO, and RE strains were grown in YPD medium at 37°C, harvested, lyophilized, and subjected to analysis. Contents of SGs and ASGs extracted from conidia (corresponding to 1 μg of conidial protein) or 4 mg of dried hyphae were quantified using LC-ESI MS/MS. Data are presented as Mean ± SD (n = 3). DCW, dry cell weight. (TIF) [file ppat.1013089.s009.tif]

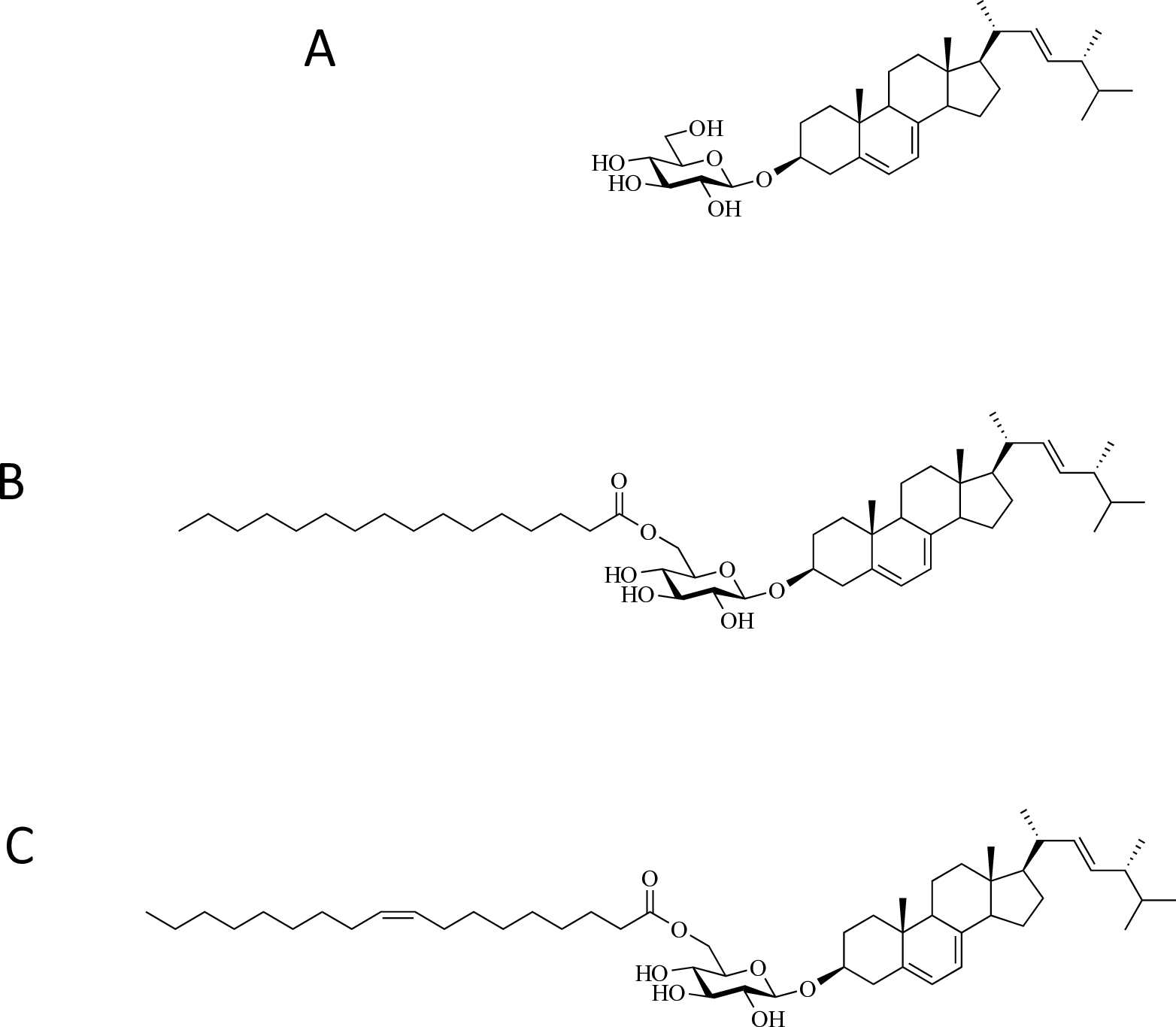

Supplement: S10 Fig — A, Ergosteryol β-D-glucopyranoside (EG); B, Ergosterol 6-O-palmitoyl-β-D-glucopyranoside (16:0-EG); C, Ergosterol 6-O-oleoyl-β-D-glucopyranoside (18:1-EG). The methods for synthesis of EG and AEGs are described in Materials and methods. (TIF) [file ppat.1013089.s010.tif]

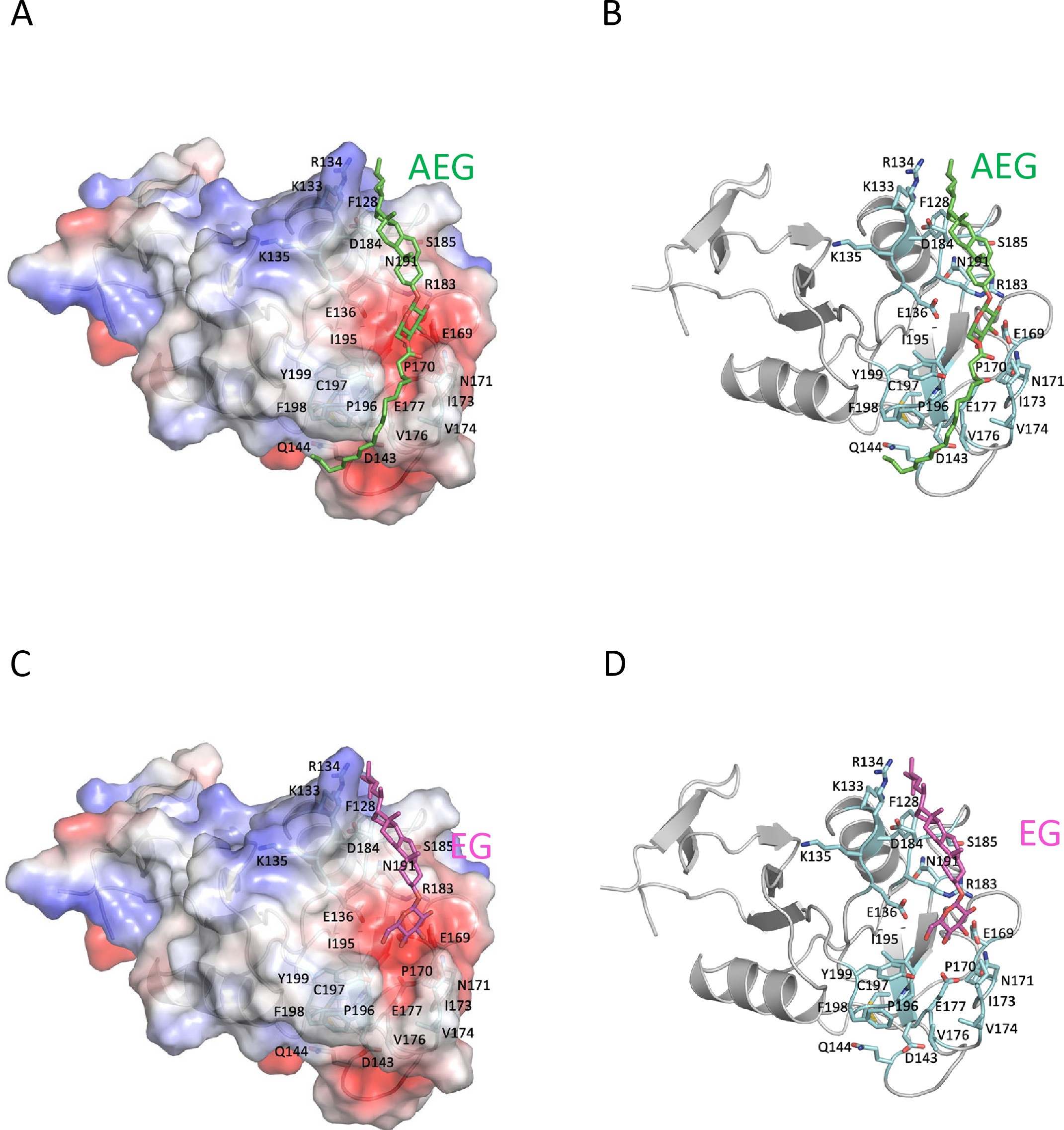

Supplement: S11 Fig — A, B, AEG-docking model (18:1-EG, colored in green); C, D, EG-docking model (EG, colored in purple). The generation of docking models was performed according to the method described in Materials and methods. The Alphafold2 model of mouse Mincle was used in its apo state (lacking Ca2+ and ligand binding) and was superimposed on the bovine Mincle structure (PDB ID: 5KTH) after truncating the first 62 N-terminal residues. In panels A and C, the surface of the Mincle is colored white, red, and blue to represent hydrophobic, negative, and positive regions, respectively. (S11) [file ppat.1013089.s011.tif]

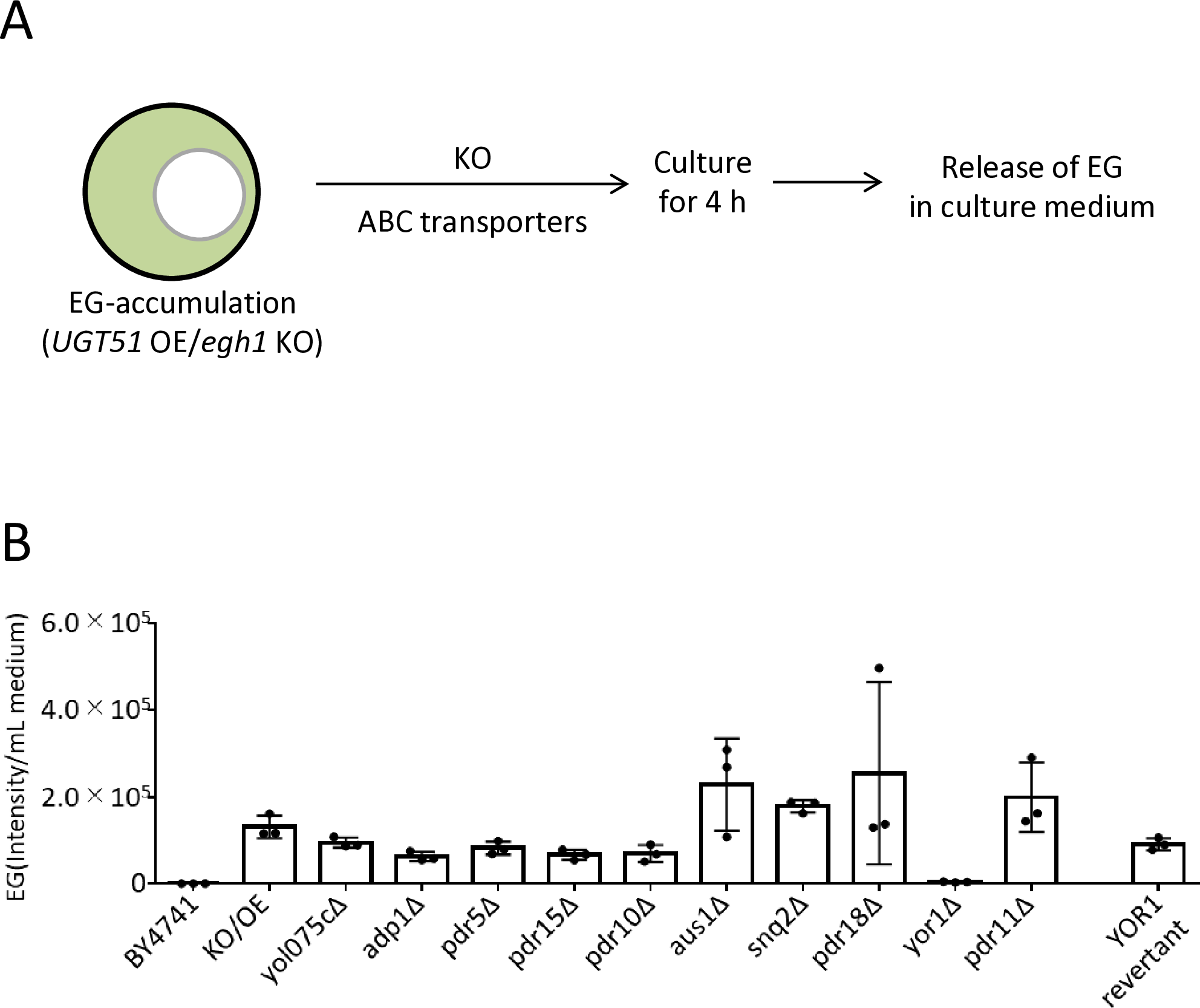

Supplement: S12 Fig — A, Experimental design. B, Quantification of EG in the medium by LC-ESI MS/MS. The control strain (KO/OE) was generated by overexpression of UGT51 [31] and disruption of EGH1 [32] in S. cerevisiae. The test strain was generated from KO/OE by disrupting each ABC transporter. The YOR1 revertant was generated by reintroducing YOR1 into a YOR1-disrupted mutant (yor1Δ). Data are presented as Mean ± SD (n = 3). (TIF) [file ppat.1013089.s012.tif]

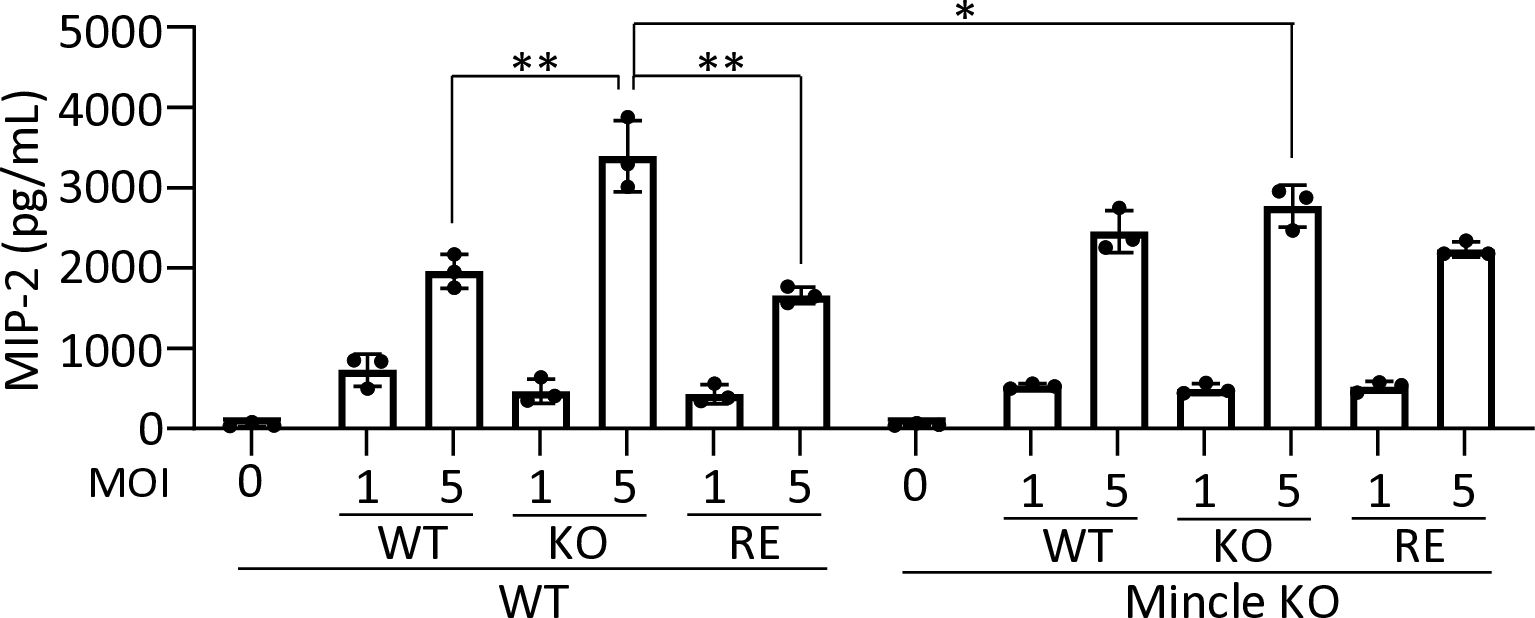

Supplement: S13 Fig — BMDCs derived from WT and Mincle KO mice were co-cultured with Cn at a multiplicity of infection (MOI) of 1 and 5 in RPMI-1640 medium containing 10% FBS at 37°C under 5% CO2 for 40 hours. The initial BMDC cell density was 1x10^5^ cells/well. RPMI-1640 medium without penicillin and streptomycin was used to prevent the inhibitory effects of antibiotics. MIP-2 production was measured by ELISA, as described in Materials and methods. Data are presented as Mean ± SD (n = 3). (TIF) [file ppat.1013089.s013.tif]

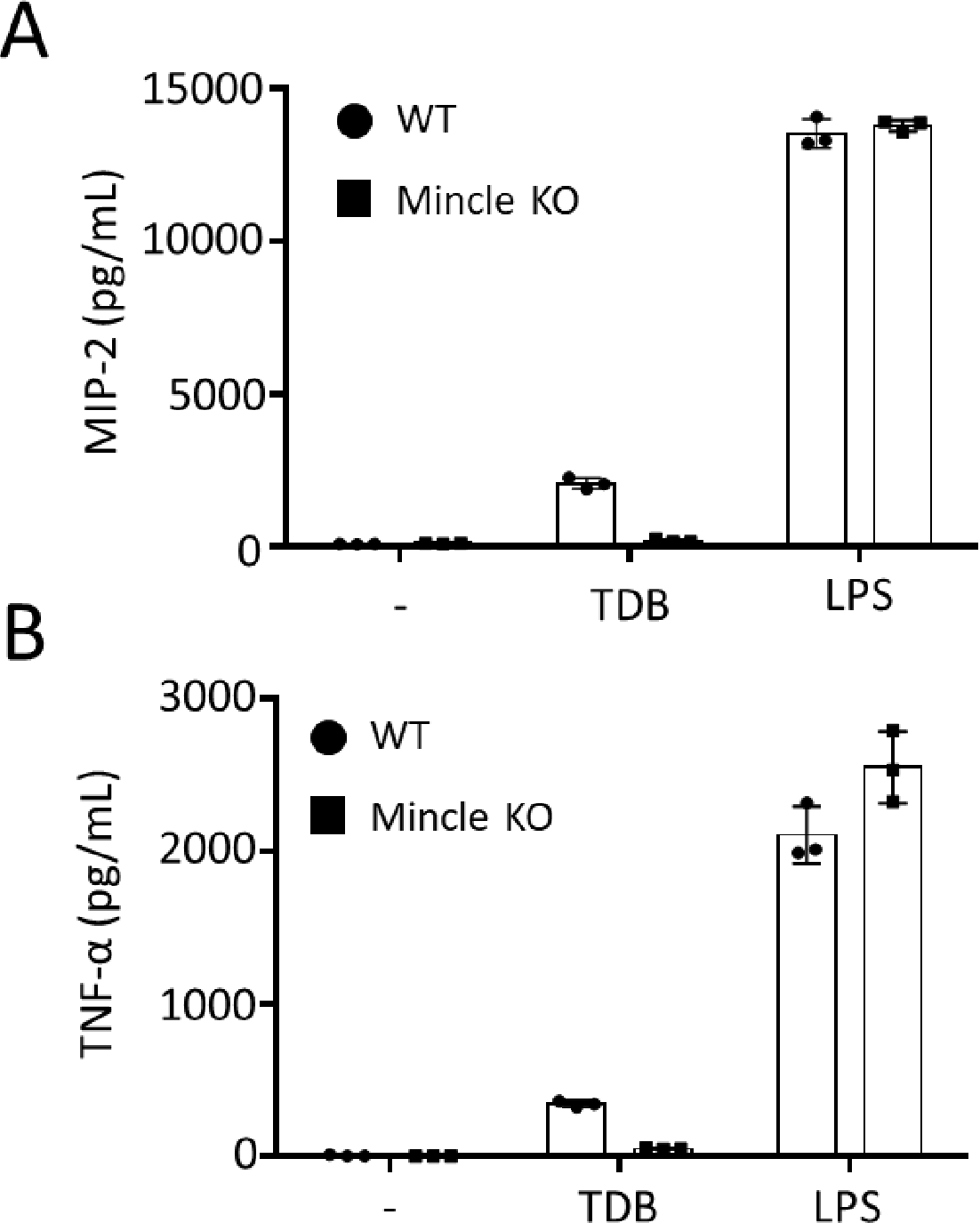

Supplement: S14 Fig — MIP-2 (A) and TNF-α (B) productions in mouse BMDCs by TDB and LPS. BMDCs from WT and Mincle KO mice were exposed to TDB (0.1nmol per well) and LPS (10ng per well) at 37°C for 40 h. ELISA was performed to determine MIP-2 and TNF-α production using specific antibodies described in Materials and methods. Data are presented as Mean ± SD (n = 3). (TIF) [file ppat.1013089.s014.tif]

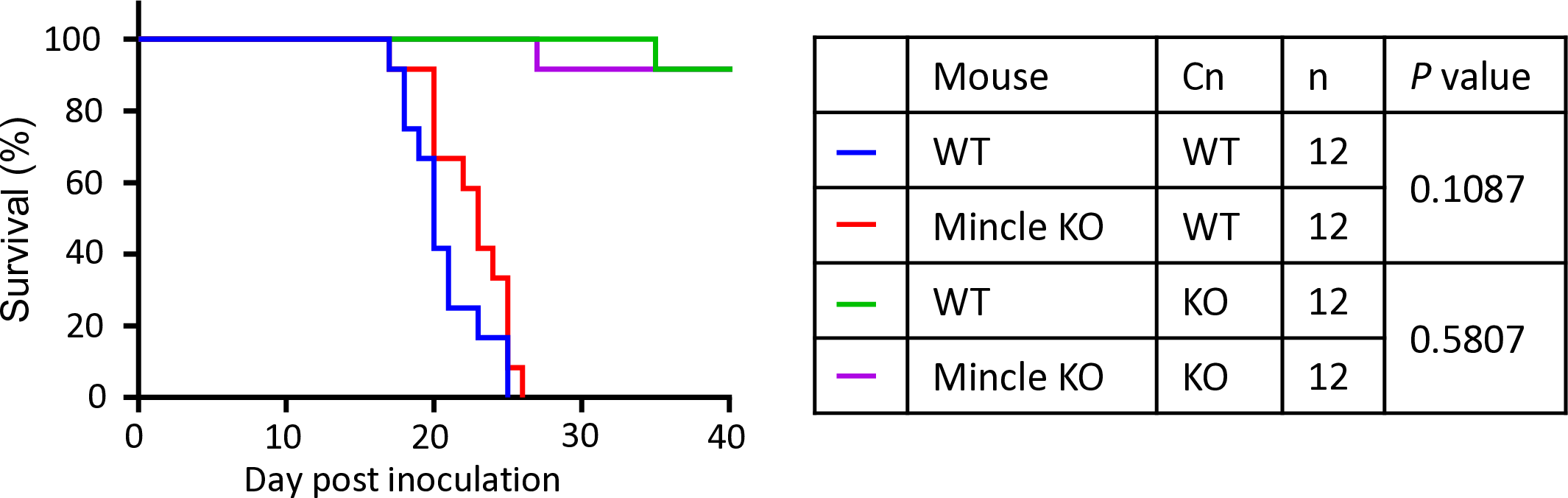

Supplement: S15 Fig — WT and Mincle KO mice (6 per group) were infected intranasally with 5 × 10^5^ WT and KO strain cells. Mice were monitored twice daily, and those showing signs of distress or becoming moribund were humanely sacrificed using carbon dioxide. The experiments were independently repeated twice, confirming reproducibility. The combined results were subjected to statistical analysis. (TIF) [file ppat.1013089.s015.tif]

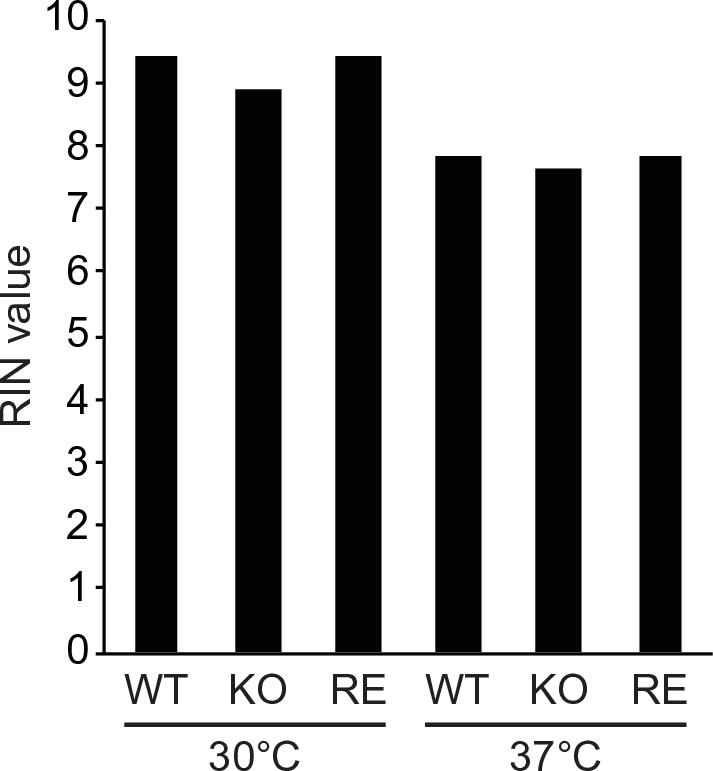

Supplement: S16 Fig — RNAs were prepared from WT, KO, and RE strains cultured in YPD medium at 30 and 37°C for 3 days following the methods described in Materials and methods. RNA integrity was assessed using Agilent Bioanalyzer 2100 (Agilent Technologies), with RNA Integrity Numbers (RIN) measured for each sample. All samples had RIN values above 7, indicating that they were suitable for RNA sequencing and qPCR. (TIF) [file ppat.1013089.s016.tif]
