# Supplementary material for: Vacuolar sterol β-glucosidase EGCrP2/Sgl1 deficiency in Cryptococcus neoformans: Dysfunctional autophagy and Mincle-dependent immune activation as targets of novel antifungal strategies
Source: PLoS Pathog. 2025 Apr 24;21(4):e1013089. doi: 10.1371/journal.ppat.1013089 (PMC12061408; doi:10.1371/journal.ppat.1013089)
Supplement: Attachment — Submitted filename: Response to Reviewers Red.pdf [file ppat.1013089.s022.pdf]

Response to Reviewers' Comments and Questions

We sincerely thank the editor and reviewers for their valuable suggestions and insightful comments. While we could not perform all the additional experiments requested, we believe that the experimental results we obtained sufficiently addressed their concerns. For clarity, we have provided responses to each reviewer's questions and comments, highlighted in blue. Revised or newly added statements are marked in red in the revised manuscript. We hope that the revisions made in response to the editor's and reviewers' feedback have improved the manuscript and that it is now suitable for publication in *PLOS Pathogens*.

Part I - Summary

Reviewer #1: In this study from Watanabe et al, the authors aim to understand the mechanisms underlying decreased virulence that has been previously observed in Cryptococcus neoformans strains deficient for sterol-beta-glucosidase. The authors suggest a two-fold mechanism: 1) Sgl1-deficient Cn results in increased rates of yeast cell death when cultured at 37C due to defective autophagy, and 2) Deletion of Sgl1 results in accumulation of ergosterol-beta-glucoside as wells as acetylated ergosterol-beta-glucoside, the latter of which can function as a ligand for the C-type lectin receptor Mincle when secreted by EVs. Overall, the results demonstrating defective autophagy in vitro and accumulation of EGs and AEGs are clear. The identification of AEGs as Mincle ligands is also an important insight.

However, the significance of the autophagy defect vs Mincle activation in vivo, and the actual activation of Mincle by the KO Cn strain are less convincing. The authors generally rely on ultracentrifugation of supernatants to isolate EVs and then test their ability to activate DCs, however, this approach may result in non-physiological enrichment of trace amounts of lipid activity.

Thank you for your comments. Any concerns or questions raised by the reviewer will be answered in response to individual questions.

Major Comments:
1. The authors show clearly that 37C culture of KO Cn results in increased yeast cell death and that this correlates with decreased expression of autophagy genes and localization of ATG8 with vacuoles. What is less clear to me is whether the observed effects on autophagy are a cause or a consequence of the cell death at 37C?

Thank you for raising this important question. To address the reviewer's question of whether autophagy is a cause or a consequence of KO Cn cell death, we conducted a time-course analysis comparing the appearance of dead cells in KO Cn with WT and RE cultured in SD-N (nitrogen starvation). Our results showed that in SD-N medium, KO Cn exhibited a significantly higher number of dead cells at 4 and 8 hours compared with WT and RE (Fig 7). Under the same conditions, autophagy was induced in WT and RE at 1-2 hours, while KO Cn failed to induce autophagy until 4 hours (Fig 5). Conversely, in YPD medium, KO Cn did not show cell death until 8 hours of culture (Fig 7). These findings indicate that autophagy dysfunction of KO Cn occurs under nitrogen starvation at 37°C, and it precedes cell death. We have added Fig 7 and its description (lines 235-239, 558-560, 708-719) to the revised manuscript.

2. Much of the analyses done for testing autophagy function are done in nitrogen starvation media – how does this relate to the late increase in cell death when culturing in YPD?

To address the reviewer's concern, we investigated autophagy induction in KO Cn cultured in YPD medium (Fig 4). The results demonstrated that from day 3, autophagy was successfully induced in WT and RE Cn, whereas only minimal autophagy was observed in KO Cn. This impaired autophagy induction in KO Cn ultimately leads to a significantly higher number of dead cells observed on day 3 compared with WT and RE Cn when cultured in YPD medium at 37°C (Fig 2B-D). We have added Fig 4 and its description (lines 215-222) to the revised manuscript.

3. Are there ways to rescue/increase autophagy in the KO cells to see if this rescues?

To our knowledge, the most effective condition to induce autophagy is nitrogen starvation, which is achieved by culturing in SD-N medium. Under these conditions, we observed minimal autophagy induction in KO Cn. Based on our understanding, no additional reagents or methods could further enhance autophagy induction in KO Cn under nitrogen starvation. Currently, EGCrP2/Sgl1 gene expression is the only way to rescue autophagy defects in KO Cn.

4. I'm convinced that KO Cn produces more EG and AEGs, and that AEGs will activate Mincle when you add them to BMDCs or into reporter lines. My concern with the EV experiments is that these may be added in supraphysiological concentrations in the functional assays. In Fig 12, the KO EVs give only a 2-fold increase in production of MIP-2 from BMDCs compared with WT, which is inconsistent with almost non-existent concentrations of EG and AEGs in WT Cn compared to KO. As a very simple experiment, if you just challenge BMDCs or the Mincle reporter line with WT vs KO Cn, do the KO cells actually secrete enough AEG to activate? If so, can you observe the same effect with conditioned supernatants? These sorts of experiments are crucial to perform before doing ultracentrifugation on supernatants.

Thank you for your comments. The high level of MIP-2 production promoted by WT EVs in BMDCs may be attributed to the presence of substances within the EVs that are responsive to CLRs or other immunoreceptors. Previous studies have reported that Cn EVs can enhance cytokine production in murine dendric cells (Clara Luna Marina et al, Nutritional Conditions Modulate C. neoformans Extracellular Vesicles' Capacity to Elicit Host Immune Response. Microorganisms 2020, 8, 1815; doi:10.3390/microorganisms8111815), which may explain the elevated baseline of cytokine production observed with WT EVs. This elevated baseline could reduce the apparent difference in MIP-2 production between WT and KO EVs (Fig 14A, B).

We acknowledge the reviewer's suggestion to directly challenge BMDCs or the Mincle reporter assay system with KO Cn or conditioned supernatants. We compared the production of MIP-2 in BMDCs from WT and Mincle KO mice when co-cultured with WT, KO, and RE Cn. The results showed that the production of MIP-2 was significantly higher with KO Cn than with WT and RE Cn. Additionally, the increase in MIP-2 production by KO Cn was significantly suppressed when BMDCs from Mincle KO mice were used (S Fig 12). These results indicate that KO Cn activates BMDCs when co-cultured to produce cytokine in a Mincle-dependent manner. We have added S12 Fig and its description (lines 387-390) and a discussion about it (lines 532-538) to the revised manuscript.

5. Can the authors perform some experiments to assess the relative contributions of autophagy defects vs increased Mincle production to the virulence defect in vivo? The authors show an increase in CFU of KO Cn at Day 3 post-infection in Mincle-deficient mice compared to WT mice. This is a crucial line of experiments in that it asks how much of the in vivo defect is due to increased Mincle ligand production vs autophagy defects – I think the manuscript would be well-served to do this experiment as a survival curve or CFU time course to see how much the infection outcome is altered in Mincle KO mice. Alternatively, since Card9 deletion has a much stronger phenotype in vitro for loss of activation in response to AEGs, it might be better to see how much of the CFU defect of Sgl1KO Cn is rescued in Card9-/- mice.
Thank you for your suggestions. To address the reviewer's concerns about the relative contributions of autophagy defects vs increased Mincle production to the virulence defect in vivo, we performed Cn infection experiments using WT and Mincle KO mice. In our current mouse infection model, KO Cn lost virulence regardless of the presence or absence of Mincle (S14 Fig). In the mouse infection model, KO *Cn* CFU in the lungs of WT mice on day 3 is less than 5% compared with WT and RE *Cn* CFU (Fig 1B). These findings indicate that the autophagy defect in KO Cn is the primary reason for its loss of virulence. We have added S14 Fig, its description (lines 412-414), a discussion of it (lines 469-481), and a statement to the Abstract (lines 49-50).

Previous studies in a mouse model of *Candida albicans* infection have shown that Mincle deficiency alone does not significantly affect survival time of mice (Thompson A, Davies LC, Liao C Te, da Fonseca DM, Griffiths JS, Andrews R, et al. The protective effect of inflammatory monocytes during systemic C. albicans infection depends on collaboration between C-type lectin-like receptors. PLoS Pathog. 2019;15(6). https://pubmed.ncbi.nlm.nih.gov/31242262/). However, survival was markedly reduced when multiple CLRs, such as Mincle/Dectin-1, Mincle/Dectin-2, or Mincle/Dectin-1/Dectin-2, were simultaneously knocked out, indicating that other CLRs can compensate for the loss of Mincle. Similarly, the AEG-dependent immune responses observed in our study suggest that unidentified CLRs or non-CLR pathways may also play a role in compensating for the loss of Mincle (Fig. 14C, D, G). This compensatory mechanism likely explains at least in part why Mincle-deficient mice showed no significant difference in survival time compared with wild-type mice in the KO Cn infection model. The revised manuscript added these statements and a reference to the Discussion (lines 482-489).

6. Along the same lines as above, the ASG treatment along with WT Cn infection is a nice experiment in that it tests how much of an effect Mincle activation alone has on CFU in the absence of an autophagy defect. However, the actual result is not very convincing. Was this just done once? This experiment warrants a repeat and looking at later time points (if you give repeated ASG doses).

Thank you for your comments. As discussed in item 5, the primary reason KO Cn loses virulence is thought to be the inability of KO Cn to perform autophagy under starvation at 37°C. However, the experiment shown in Fig 14 suggests that the ASG-Mincle immune activation system still functions in terms of increased cytokine production and Cn clearance even when WT Cn is administered with ASiG. This result indicates that Mincle activation by ASGs can enhance immune responses without autophagy defects. This description has been added to the Discussion in the revised manuscript (lines 472-475).

The experiment was performed twice and yielded consistent results, with one dose of ASiG administered per experiment. The legend in Fig 14 has been corrected for clarity (lines 1320-1321), as the previous version was unclear and potentially misleading.

Minor comments:
7. The OD600 defects actually seem more pronounced at 25C and 30C (at least at day 7) compared to 37C. The authors suggest that this may be due to leakage of cellular contents at 37C. However, they also observe very small increased in dead Cn at 25 and 30, despite the strong OD600 defect. Can they comment on this?

Fig 2B and C show the results on day 3 of culture at 37°C, while S5 Fig shows that, by day 7, dead cells (as indicated by phloxine B staining) of KO Cn increased even at 25°C and 30°C.

8. For the model in Fig 14, I understand some of this is linking in previously published data. However, for this specific manuscript, I think the authors should remove the images from the model that are not specifically based on experiments shown in this body of work. For example, the link to gamma delta T cells is entirely speculative.

The reviewer's point is very valid, so we have surrounded the previous study with dotted lines in Fig 16 to indicate that it is not directly related to the present study. This figure now focuses on summarizing our current findings, which complement the earlier work by Del Poeta, while making clear the distinction between the two. Both studies highlight the significance of EGCrP2 (Sgl1) as a crucial antifungal target. A statement was added to the legend of Fig 16 in the revised manuscript (lines 1331-1332).

Reviewer #2:

9. In general, this paper is a succession of explorations related to the EGCrP2/Sgl1 KO mutant. I fell that there is not much link between the different parts. For example, the autophagy is not really related to the other explorations such as immunology and then EVs. We are missing here a united line for the message of this paper.

Thank you for your comments. In KO Cn, the accumulation of EG/AEGs in vacuoles leads to autophagy defects and triggers the extracellular efflux of EG/AEGs via EVs. This, in turn, activates the Mincle-mediated innate immune system. Thus, while the study covers different aspects such as autophagy, immunology, and EVs, these events are interconnected, all stemming from the vacuole-localized EGCrP2/Sgl1 defects. This integrated mechanism links the various parts of the paper, contributing to the overall message that glycolipid accumulation, autophagy defects, and immune activation are all consequences of EGCrP2 dysfunction.

Reviewer #3:

10. In this very nice study, the authors investigated the mechanisms responsible for the loss of virulence of a slg1delta mutant in Cryptococcus neoformans. This gene has been identified by this group and that of M. del Poeta, and this deletion has been found to be associated with the accumulation of ergosterol beta-glucoside (EG), but the reason for the loss of virulence remained unknown. In this paper, they showed that Slg1 accumulates in the vacuole and controls autophagy gene expression and protein localization. The authors used a battery of assays to show that a concomitant accumulation of EG acetylated derivatives (AEGs) is probably more

important than that of EG. They presented convincing experiments showing that AEGs bind to the C-type lectin receptor Mincle in a very affine manner, activating the production of cytokines that ultimately trigger the elimination of the yeast pathogen. They confirmed the importance of both this lipid and the receptor using animal models coupled with AEGs treatments. It is a very nice story and a very convincing set of data.

We sincerely thank the reviewer for the positive and thoughtful feedback. Our research aimed to clarify the mechanisms underlying the loss of virulence in the KO Cn (sg1Δ mutant), and we appreciate the reviewer's recognition of the importance of our findings regarding the autophagy defect due to the accumulation of EG and AEGs in vacuoles, activation of Mincle, and the subsequent immune responses. We hope this work contributes to a better understanding of the role of glycolipid metabolism in fungal virulence and host immunity.

Part II – Major Issues: Key Experiments Required for Acceptance
Please use this section to detail the key new experiments or modifications of existing experiments that should be absolutely required to validate study conclusions.

Generally, there should be no more than 3 such required experiments or major modifications for a "Major Revision" recommendation. If more than 3 experiments are necessary to validate the study conclusions, then you are encouraged to recommend "Reject".

11. Challenge WT vs Mincle-/- vs Card9-/- BMDCs with WT vs KO Cn live yeasts to see if the KO strain produces enough AEGs to activate the Mincle pathway.

We acknowledge your valuable suggestions. As mentioned in answer #4, we compared the production of MIP-2 in BMDCs from WT and Mincle KO mice when co-cultured with WT, KO, and RE Cn. The results showed that the production of MIP-2 was significantly higher with KO Cn than with WT and RE Cn. Additionally, the increase in MIP-2 production by KO Cn was significantly suppressed when BMCDs from Mincle KO mice were used (S12 Fig). These results suggest that the amount of AEG produced by co-cultured KO *Cn* is sufficient to activate BMDCs to produce cytokines in a Mincle-dependent manner under experimental conditions.

12. Infect Mincle-/- and/or Card9-/- mice with WT vs KO Cn and perform either survival curves or time course (day 3, 7, 10, 14) CFUs

Thank you for your valuable suggestions. As shown in answer #5, we performed Cn infection experiments using WT and Mincle KO mice. In our current mouse infection model, KO Cn lost

virulence regardless of the presence or absence of Mincle (S14 Fig). This finding strongly suggests that the autophagy defect in KO Cn is the primary reason for its loss of virulence.

13. Infect WT vs Mincle-/- mice with WT Cn along with ASGs and perform time course CFUs.

We thank the reviewer’s suggestion. Due to a limited number of available Mincle KO mice, we could not perform the requested infection experiments with WT Cn and ASGs in Mincle KO mice over a time course. However, we conducted Cn infection experiments with WT and Mincle KO mice (S14 Fig) and additional autophagy experiments (Fig 4 and Fig 7). They provide insights into the reviewer’s question regarding the relative contributions of increased Mincle ligand production versus autophagy defects in vivo.

Reviewer #2:

14. All the experiments are performed after 3 days in YPD with agitation. The cells are at this step starved and so not in a normal metabolism. Is it really physiological and do the findings could be generalized to LOG or stationary phase?

We appreciate the reviewer’s comment regarding the physiological relevance of the experimental conditions. It is generally accepted that when cultured in YPD medium with or without agitation, microorganisms pass through the log phase, enter the stationary phase, and then proceed into the death phase as nutrients are depleted. As shown in Fig 2A, the growth pattern of Cn in our study aligns with this typical progression (log, stationary, and death phases), indicating that the cells are undergoing nutrient depletion similar to what is seen in natural conditions. Del Poeta's group also investigated the growth of Cn on the YPD agar medium, and they found that KO Cn could not grow at 37°C (Pereira de Sa N, Taouil A, Kim J, Clement T, Hoffmann RM, Burke JE, et al. Structure and inhibition of Cryptococcus neoformans sterylglucosidase to develop antifungal agents. Nat Commun. 2021 Dec 1;12(1). Their result is consistent with our observations, although both culture conditions are different (liquid culture vs agar culture). Thus, while our model does not precisely mimic every aspect of in vivo physiology, it provides valuable insights into the autophagy defects of KO Cn under conditions of nutrient depletion.

We experimented with a nitrogen-starved medium (SD-N medium) instead of YPD medium to further investigate the induction of autophagy in Cn upon nitrogen starvation. In SD-N medium, autophagy was induced in WT and RE within 1–2 hours of culture initiation. In contrast, KO Cn failed to undergo autophagy (Fig 5) and consequently began to die within 4 hours (Fig 7). The revised manuscript added new experimental results on autophagy induction and cell death in a nitrogen-starved medium (Fig 7).

15. How far Phloxin B have been validated in Cryptococcus neoformans we need controls together with the experiment (Dead control 65°C 1h, H2O2 2 to 24hrs).

Thank you for your valuable suggestion. We have performed additional control experiments to validate the use of Phloxin B staining in Cn. We used heat-killed Cn cells (treated at 75°C for 30 minutes) as a positive control. The results showed that heat-killed Cn cells were effectively stained by Phloxin B, regardless of the presence or absence of EGCrP2/sgl1. We have added S4 Fig and its description (lines 179-181) in the revised manuscript.

16. RNASeq have been perform as simplicates per condition with prevent accurate analysis of the results in particular normalization and adequate calculation of fold changes. DeSeq 2 is the best method/pipeline to analyze RNASeq and should be done like this.

Thank you for your insightful comment. In the RNA-Seq analysis conducted in this study, WT, KO, and RE Cn strains were analyzed at different temperatures (25°C, 30°C, and 37°C) and incubation periods (2 and 3 days). Due to cost constraints, only one sample could be analyzed for each condition. However, we want to clarify that our RNA-seq analysis was primarily intended as a screening tool to identify potential candidate genes for further validation. Following this initial screening, all significantly differentially expressed genes in KO Cn compared to WT/RE Cn were validated through quantitative qPCR analysis (n=3) using multiple reference genes according to the reviewer's suggestion.

Regarding the use of DESeq2, we appreciate the recommendation and attempted to analyze the RNA-seq data using this tool. However, we encountered challenges with clustering that limited its applicability in our specific dataset. While DESeq2 is widely recognized as a robust pipeline for RNA-seq analysis, the limitations in clustering results prompted us to explore alternative approaches better suited to our study.

17. Relative expression by qPCR should not use 2-ΔΔCq as individual qPCR have a different efficiency and should use at least 3 reference genes (see 1. Vandesompele J, Preter K de, Pattyn F, Poppe B, Roy N van, Paepe A de, Speleman F. Accurate normalization of real-time quantitative RT-PCR data by geometric averaging of multiple internal control genes. Genome biology. 2002 Jun 18;3(7):RESEARCH0034.)

Thank you for your valuable suggestion. In the revised manuscript, we reanalyzed the relative mRNA expression levels using the PCR efficiency-corrected method (Pfaffl MW, *Nucleic Acids Research*, 2001, e45, https://doi.org/10.1093/nar/29.9.e45) instead of the 2-ΔΔCq method. This approach normalizes relative mRNA expression with two reference genes, ACT1

(Actb) and GAPDH, as internal controls. The results, presented in Fig. 3C and Fig. 15E of the revised manuscript, demonstrate that normalization with either ACT1(Actb) or GAPDH produced consistent outcomes, confirming the reliability of our qPCR data. The revised manuscript added the statement (lines 666-667). We appreciate your input and have revised the manuscript accordingly to enhance the robustness of our analysis.

18. Considering that more than 60% of the cell population is dead (Fig 2C), it is difficult to make sense of the transcriptome analysis of the cells cultivated in the same condition. Are the ATG8 transcript decreased because of death? I would assume so. Probably the half-life of those transcript are much lower than that of the reference gene explaining this decrease in the expression.

Thank you for your insightful comments. To ensure RNA quality, we have assessed RNA Integrity Numbers (RIN) for WT, KO, and RE *Cn* cultured at 30°C and 37°C. All samples had RIN values above 7.0, indicating minimal RNA degradation and sufficient quality for RNA-seq and qPCR analysis. Therefore, the observed decrease in *ATG8* transcript levels is unlikely to be an artifact of RNA degradation from dead cells but rather a biologically relevant effect. Furthermore, some KO *Cn* genes were more upregulated than WT and RE at 37°C in our RNA-seq data (Fig. 3A, cluster C), addressing the concern that KO *Cn* genes might appear under-expressed due to RNA breakage. We have added S15 Fig and its description to the revised manuscript (lines 648–651, 662).

19. Enrichment score is between 1 and 1.5 which is not high. Then, is it really meaningful?

The enrichment score for autophagy is between 1 and 1.5, which is relatively low as suggested by the reviewer. However, autophagy forms a distinct cluster and is positioned as one of the top biological function terms, which suggests its importance in the biological context of cell death in KO Cn at 37°C.

20. I am not sure that the finding on fumigatus are really bringing something to the message on cryptococcus (Aspergillus ascomycetes and Cryptococcus basitiomycetes are indeed very different organisms in terms of biology.)

Thank you for your comments. There are two key points regarding the inclusion of A. fumigatus data. First, previous studies have shown that EGCrP2/Sgl1 KO A. fumigatus is lethal, but those studies only reported the accumulation of EG. In contrast, this study is the first to demonstrate that A. fumigatus also accumulates AEG, similar to Cn. This shows that the loss or inhibition of EGCrP2/Sgl1 in pathogenic fungi with EGCrP2/Sgl1, such as Cn and A. fumigatus, leads to the

accumulation of not only EG but also AEG. Second, the WHO recognizes A. fumigatus and Cn as a significant fungal pathogen. As the manuscript focuses on potential new antifungal targets, the data on A. fumigatus provides a valuable supplementary context.

Reviewer #3: The following comments are intended to improve the quality of this manuscript.

21. The use of Phloxin B staining to determine cell viability needs to be validated. In the image shown (Figure 2B), both mother and budding cells are stained, which is strange for a dying cell.

Thank you for your valuable suggestion. In the revised experiments, we performed control experiments to validate Phloxin B staining for detecting dead Cn cells. The results confirmed that Phloxin B stains dead cells (S4 Fig). Based on these findings, it is reasonable to assume that, in this case, both the mother and daughter cells are dead, as both are stained.

22. Figure 5 shows not only degradation but also production of Atg8, which, according to the qPCR experiments, also decreases over time. This needs to be better explained.

Fig 5 is a GFP-Atg8 overexpression experiment and does not represent endogenous Atg8 expression as measured by qPCR.

23. Sgl1 localization (Figure 6): showing one cell at high magnification is good, but it should be accompanied by an image showing multiple cells to convince the reviewer that this co-localization is representative.

Thank you for your suggestion. In the revised manuscript, we have included additional images (S7 Fig) showing multiple cells at a lower magnification to provide more representative evidence of the EGCrP2/Sgl1 localization in the vacuoles.

24. Line 280 "significantly higher should be confirmed by a statistical test and a p-value should be given.

Thank you for your suggestion. We have performed the statistical test and included the corresponding p-value in Figure 10 of the revised manuscript.

25. The paragraph starting on 309 describing the mouse mincle docking models with AEG or AG is interesting, but the conclusion would need more experiments to be confirmed. The calcium implication comes out of nowhere for the reader. Its implication and role could be confirmed experimentally. This paragraph could be deleted as well.

Thank you for your comment. It is well-known that C-type lectins require calcium for binding to sugars. However, our discussion on the stability of the interaction with the current ligands is novel and significant. As demonstrated by last year's Nobel Prize in Chemistry, advancements in AI-driven protein modeling, such as AlphaFold, have greatly improved the accuracy of protein structure and ligand binding predictions. As pointed out by the reviewer, however, crystallography of the ligand-receptor complex would be essential to draw firm conclusions, and we have added this point to the revised manuscript (lines 466-468).

26. Same for the next paragraph. The point mutation mutant should be tested if one wants to conclude.

We fully agree with the reviewer's comment. To reach a definitive conclusion, the co-crystallization analysis of the ligand and Mincle is crucial, and we have added this to the revised manuscript (lines 466-468).

27. Figure 11C, E, F: EG and AEG are detectable in WT and RE strain. This is surprising as it was stated earlier that it is not detectable in the WT cell. This should be discussed.

Thank you for your comment. While EG and AEG were hardly detectable in WT cells by TLC, they were detectable by LC-MS. We have modified the manuscript to reflect this distinction and have revised "was not detectable" to "was hardly detectable by TLC" for clarity (lines 257).

28. Line 368: There is probably a word missing in this sentence and in the next one, since EVs do not produce MIP-2 or IL-8.

Thank you for the indication. We have corrected the sentence as follows: " The increase in MIP-2 production in mouse bone marrow-derived dendric cells (BMDCs) by EVs from KO *Cn* is significantly higher than that from WT and RE *Cn* (Fig 14A) (lines 384-385)."

Part III – Minor Issues: Editorial and Data Presentation Modifications
Please use this section for editorial suggestions as well as relatively minor modifications of existing data that would enhance clarity.

Reviewer #1: N/A

Reviewer #2: (No Response)

Reviewer #3: none

PLOS authors have the option to publish the peer review history of their article (what does this mean?). If published, this will include your full peer review and any attached files.

If you choose "no", your identity will remain anonymous but your review may still be made public.

Do you want your identity to be public for this peer review? For information about this choice, including consent withdrawal, please see our Privacy Policy.

Reviewer #1: No

Reviewer #2: No

Reviewer #3: No

[NOTE: If reviewer comments were submitted as an attachment file, they will be attached to this email and accessible via the submission site. Please log into your account, locate the manuscript record, and check for the action link "View Attachments". If this link does not appear, there are no attachment files.]

Figure resubmission:
While revising your submission, please upload your figure files to the Preflight Analysis and Conversion Engine (PACE) digital diagnostic tool, https://pacev2.apexcovantage.com/. PACE helps ensure that figures meet PLOS requirements. To use PACE, you must first register as a user. Registration is free. Then, login and navigate to the UPLOAD tab, where you will find detailed instructions on how to use the tool. If you encounter any issues or have any questions when using PACE, please email PLOS at figures@plos.org. Please note that Supporting Information files do not need this step. If there are other versions of figure files still present in your submission file inventory at resubmission, please replace them with the PACE-processed versions.

Reproducibility:
To enhance the reproducibility of your results, we recommend that authors of applicable studies deposit laboratory protocols in protocols.io, where a protocol can be assigned its own identifier (DOI) such that it can be cited independently in the future. Additionally, PLOS ONE offers an

option to publish peer-reviewed clinical study protocols. Read more information on sharing protocols at https://plos.org/protocols?utm_medium=editorial-email&utm_source=authorletters&utm_campaign=protocols

Additional Editor Comments :
The concerns regarding controls raised by Reviewer 2 and 3 need to be addressed.
Both reviewers 1 and 2 were concerned about the biological relevance of the studies, and these concerns should be addressed with proposed experiments or similar ones that provide the necessary clarification.

We thank the editor for highlighting this critical point raised by the reviewers. To address the concerns regarding controls, we performed phloxine B staining experiments using preheated, dead Cn cells as a control to confirm that phloxine B stains dead cells explicitly. As detailed in the response to question #21, the results demonstrated that only dead cells were stained with phloxine B. This new data has been added to the revised manuscript as S4 Fig.

To address the reviewers' concerns about the biological relevance of our studies, we conducted Cn infection experiments using WT mice and Mincle KO mice. As described in the response to question #12 and shown in S14 Fig, the results revealed that KO Cn lost their virulence regardless of the presence or absence of Mincle. In the mouse infection model, KO Cn CFU in the lungs of WT mice on day 3 is less than 5% compared with WT and RE Cn CFU (Fig 1B). These results strongly suggest that the autophagy defect in KO Cn is the primary reason for its loss of virulence.

Furthermore, to strengthen this conclusion, we performed a time-course analysis comparing the appearance of dead cells in KO, WT, and RE Cn cultured in SD-N medium. As outlined in the response to question #1, the results demonstrated that autophagy dysfunction in KO Cn under nitrogen starvation at 37°C precedes cell death. This supports the hypothesis that defective autophagy is the underlying cause of cell death in KO Cn.

We hope these additional experiments and findings satisfactorily address the reviewers' concerns.
