# Supplementary material for: Vacuolar sterol β-glucosidase EGCrP2/Sgl1 deficiency in Cryptococcus neoformans: Dysfunctional autophagy and Mincle-dependent immune activation as targets of novel antifungal strategies
Source: PLoS Pathog. 2025 Apr 24;21(4):e1013089. doi: 10.1371/journal.ppat.1013089 (PMC12061408; doi:10.1371/journal.ppat.1013089)
Supplement: Attachment — Submitted filename: Response to Reviwer.docx [file ppat.1013089.s023.docx]

Kirsten Nielsen, Ph. D

Guest Editor

PLOS Pathogens

Dear Dr. Nielsen,

We appreciate your review of our resubmitted manuscript and helpful suggestions for improvement. As suggested by the editor and Reviewer 3, we have revised the manuscript to address the concerns raised by Reviewer 3. The comments and suggestions from the editor and Reviewer 3 are numbered in order and are shown in blue, while our responses are in black.

Editor:

1) Move the RNAseq figure(s) to supplemental data and clearly state in the manuscript that the studies were preliminary and had only a single replicate.

We have moved the RNAseq data to the Supplementary Data as S6 Fig and mentioned in the text that these studies are preliminary (line lines 120-122; 192). The figure legend for S6 Fig indicates that the data represent a single replicate (lines 1382-1384).

2) Address Reviewer 3's concerns regarding the validity of the results from the PhloxinB results based on the current studies. An additional experiment may be warranted.

We have included a thorough explanation in our response to Reviewer 3.

3) The experiment to validate the PhloxinB assay as a means of assessing cell viability is not the one expected. The author should sort out the PhloxinB positive cells and plate them to check that they are dead. Again, the fact that both the mother cell and the bud can be positive suggests that the assay is not valid.

Thank you for your comments and suggestions. Reviewer 3 proposed sorting Phloxin B-positive cells and plating them to verify their viability. Under our experimental conditions, we observed that 100% of *Cryptococcus neoformans* (*Cn*) cells became Phloxine B positive after heat treatment at 75°C for 30 minutes (S4 Fig A), and none of these cells formed colonies on YPD agar plates, regardless of whether SGL1 was present or absent (Heat, S4 Fig B). Since all cells were Phloxine B positive (S4A Fig), additional sorting was unnecessary, as every plated cell was already identified as Phloxine B positive. Conversely, Phloxine B-negative cells formed colonies (Control, S4 Fig B). These results demonstrate that Phloxin B-positive cells are non-viable, meeting the general criteria for a control experiment, in which Phloxin B stains dead cells but not living ones. To clarify the relationship between each operation and colony formation, we indicated the respective boundaries with dashed lines in S4 Fig B in the revised manuscript. The statement about the control experiment has been added to the text (lines 178-181).

Reviewer 3 is concerned about the use of Phloxine B for determining cell viability, particularly because both mother and daughter cells are stained. However, prior studies have established that Phloxine B is a well-validated reagent for staining dead cells in yeast research (1–9). Stolp et al. thoroughly examined the relationship between cell death and staining with Phloxine B and propidium iodide (PI) in budding yeast (1). Their findings indicate that Phloxine B is more sensitive than PI in detecting dead cells. More importantly, they observed that both mother and daughter cells were stained under their experimental conditions. It is also worth noting that in *Cn*, both mother and daughter cells are stained with PI after treatment with antifungal drugs such as fluconazole (10) and isobavachalcone (11). Furthermore, nocodazole induces mitotic cell death in yeast cells, suggesting that both mother and daughter cells could die under stressful conditions (12). Thus, our observation that both mother and daughter cells are stained with Phloxine B is consistent with established findings and does not undermine the assay's validity.

The reviewer's concern may stem from our data in the YPD medium (Fig. 2B) and SD-N medium (Fig. 7), where Cn was not exposed to heat or antifungal drugs but underwent nitrogen starvation—an established stress condition. It is reasonable to expect both mother and daughter cells to become Phloxine B positive under such stress. Unfortunately, we currently lack access to a cell sorter capable of separating Phloxine B-positive from negative cells. However, based on our current data and previous studies, we believe that additional sorting experiments are unnecessary to validate Phloxine B staining as an indicator of cell death.

References

1.	Stolp ZD, Kulkarni M, Liu Y, Zhu C, Jalisi A, Lin S, et al. Yeast cell death pathway requiring AP-3 vesicle trafficking leads to vacuole/lysosome membrane permeabilization. Cell Rep [Internet]. 2022 Apr 12 [cited 2025 Mar 11];39(2). Available from: https://pubmed.ncbi.nlm.nih.gov/35417721/

2.	Minois N, Frajnt M, Wilson C, Vaupel JW. Advances in measuring lifespan in the yeast Saccharomyces cerevisiae. Proc Natl Acad Sci U S A [Internet]. 2005 Jan 11 [cited 2025 Mar 14];102(2):402–6. Available from: https://www.pnas.org/doi/abs/10.1073/pnas.0408332102

3.	Tsukada M, Ohsumi Y. Isolation and characterization of autophagy-defective mutants of Saccharomyces cerevisiae. FEBS Lett [Internet]. 1993 Oct 25 [cited 2025 Mar 14];333(1–2):169–74. Available from: https://onlinelibrary.wiley.com/doi/full/10.1016/0014-5793%2893%2980398-E

4.	Kato S, Suzuki K, Kenjo T, Kato J, Aoi Y, Nakashimada Y. Single-Cell Time-Lapse Observation Reveals Cell Shrinkage upon Cell Death in Batch Culture of Saccharomyces cerevisiae. mBio [Internet]. 2021 Dec 1 [cited 2025 Mar 14];12(6). Available from: https://journals.asm.org/doi/10.1128/mBio.03094-21

5.	Noda T. Viability assays to monitor yeast autophagy. Methods Enzymol [Internet]. 2008 [cited 2023 Aug 30];451:27–32. Available from: https://pubmed.ncbi.nlm.nih.gov/19185710/

6.	Matynia A, Mueller U, Ong N, Demeter J, Granger AL, Hinata K, et al. Isolation and Characterization of Fission Yeast sns Mutants Defective at the Mitosis-to-Interphase Transition. Genetics [Internet]. 1998 Apr 1 [cited 2025 Mar 14];148(4):1799–811. Available from: https://dx.doi.org/10.1093/genetics/148.4.1799

7.	Suzuki SW, Onodera J, Ohsumi Y. Starvation Induced Cell Death in Autophagy-Defective Yeast Mutants Is Caused by Mitochondria Dysfunction. PLoS One [Internet]. 2011 Feb 25 [cited 2024 Aug 10];6(2):e17412. Available from: https://journals.plos.org/plosone/article?id=10.1371/journal.pone.0017412

8.	Kwolek-Mirek M, Zadrąg-Tęcza R, Bednarska S, Bartosz G. Acrolein-Induced Oxidative Stress and Cell Death Exhibiting Features of Apoptosis in the Yeast Saccharomyces cerevisiae Deficient in SOD1. Cell Biochem Biophys [Internet]. 2015 Nov 14 [cited 2024 Aug 10];71(3):1525–36. Available from: https://link.springer.com/article/10.1007/s12013-014-0376-8

9.	Mizushima N, Noda T, Yoshimori T, Tanaka Y, Ishii T, George MD, et al. A protein conjugation system essential for autophagy. Nature 1998 395:6700 [Internet]. 1998 Sep 24 [cited 2025 Mar 14];395(6700):395–8. Available from: https://www.nature.com/articles/26506

10.	Chang YC, Lamichhane AK, Kwon-Chung KJ. Cryptococcus neoformans, unlike candida albicans, forms aneuploid clones directly from uninucleated cells under fluconazole stress. mBio [Internet]. 2018 Nov 1 [cited 2025 Mar 14];9(6). Available from: https://journals.asm.org/doi/10.1128/mbio.01290-18

11.	Qian W, Lu J, Gao C, Liu Q, Li Y, Zeng Q, et al. Deciphering antifungal and antibiofilm mechanisms of isobavachalcone against Cryptococcus neoformans through RNA-seq and functional analyses. Microb Cell Fact [Internet]. 2024 Dec 1 [cited 2025 Mar 14];23(1):1–21. Available from: <https://microbialcellfactories.biomedcentral.com/articles/10.1186/s12934-024-02369-2>

12. Endo K, Mizuguchi M, Harata A, Itoh G, Tanaka K. Nocodazole induces mitotic cell death with apoptotic-like features in Saccharomyces cerevisiae. FEBS Lett [Internet]. 2010 Jun 3 [cited 2025 Mar 18];584(11):2387–92. Available from: https://onlinelibrary.wiley.com/doi/full/10.1016/j.febslet.2010.04.029

4) I missed this in my first review, but having a single replicate for the RNA-Seq experiment is a real problem. I understand that the authors see this experiment as a way to identify potentially regulatory genes that would be validated by qRT-PCR. However, the description of the RNA-Seq result should be different. For example, it is possible to write and RNA-seq data analysis 'revealed' something. This paragraph should be changed and the fact that a single replicate was generated should be clearly stated in the results paragraph. The figure 3A and B should be deleted.

According to the editor and your suggestions, we moved the RNAseq data (Figs. 3A and B) to the Supplementary Data as S6 Fig. and noted in the legend that these data are a single replicate (lines 1382-1384). We also stated in the text that these studies are preliminary (lines 120-122; 192).

5) The name of the gene should be changed. The official name is SLG1, as should be a gene name in Cryptococcus (3 letters and a number). It cannot be EGCrP2/Sgl1. This should be changed in the manuscript.

Thank you for your suggestion. We changed the name of the gene encoding EGCrP2/Sgl1 to *SGL1* (Italic). On the other hand, we retained the name of the enzyme EGCrP2/Sgl1 to reflect the historical context of its discovery.

6) The strains cannot be named KO Cn or RE Cn. slg1delta should identify the mutant strain. The reconstituted strain should be named slg1delta::SLG1.

We appreciate the reviewer's suggestion regarding mutant nomenclature. To adhere to standard conventions, we have referred to the *SGL1*-deficient *Cn* as *sgl1Δ* (KO) and the reconstituted strain as *sgl1Δ::SGL1* (RE) in the abstract and upon first mention in the text. Subsequently, we have used KO and RE as abbreviations throughout the revised manuscript for clarity and conciseness. This method ensures consistency while enhancing readability, especially in the figures.

We appreciate your valuable feedback, which has contributed to the improvement of our manuscript. We hope the revised version meets the standards for publication in PLOS Pathogens.

Best regards,

Makoto Ito, Ph. D.

Professor Emeritus

Kyushu University
